# Enduring Dilemmas in Gastroenterology

**DOI:** 10.3390/diagnostics14010065

**Published:** 2023-12-27

**Authors:** Vishal Sharma

**Affiliations:** Department of Gastroenterology, Postgraduate Institute of Medical Education and Research, Chandigarh 160012, India; sharma.vishal@pgimer.edu.in

Making a correct diagnosis is the first, and most important, step in the therapeutic journey of a disease. Misdiagnosis is costly; it delays the initiation of correct therapy, exposes patients to the costs and adverse effects of unwarranted therapies, and increases the risk of complications and adverse outcomes [1]. It is, therefore, incumbent on all clinicians to make all efforts to provide the correct diagnosis as soon as possible. Alas, this often is not possible, and the path to achieving a correct diagnosis is often complex. It entails clinical suspicion based on clinical symptoms, appropriately directed investigations, a proper choice of diagnostics and their interpretation, and, lastly, the discrimination of diagnosis mimics. This Special Issue of *Diagnostics* on the **Diagnosis and Prognosis of Gastrointestinal Diseases** tackles some of these issues head-on.

One of the most enduring dilemmas in clinical medicine is the discrimination of abdominal tuberculosis from its close mimics, namely, Crohn’s disease from gastrointestinal tuberculosis, and tuberculous peritonitis from peritoneal carcinomatosis [2,3]. Two articles in the current issue address this. In an observational study by Seth R et al., the authors report on the value of perfusion computed tomography in differentiating gastrointestinal tuberculosis (GITB) and Crohn’s disease (CD) [3]. GITB and CD are close mimics, with similar clinical, endoscopic, radiological, and histological findings. The microbiological positivity, the holy grail for diagnosing GITB, is less frequent; therefore, many patients require a trial of antitubercular therapy to achieve a conclusive diagnosis [4,5]. Many methods have been described to achieve a conclusive differentiation, but achieving a clear diagnosis may still not be feasible in many cases [6,7]. Perfusion CT entails serial imaging of the region of interest after intravenous contrast injection to study the dynamics of blood flow, blood volume, and mean transit time in order to identify patterns suggestive of underlying disease [8]. In a first-of-its-kind report, the authors suggest that these parameters could help differentiate GITB and CD: active CD has a higher blood flow and permeability than GITB. Their study was limited by a small number of patients and made discriminations between active and inactive Crohn’s disease. The applicability of these findings needs to be tested in future reports; however, a report published later suggests that perfusion CT could indeed be used in response assessment in GITB [9]. Another paper deals with diagnostic confusion between tuberculous peritonitis (TBP) and peritoneal carcinomatosis (PC) and reports clinical and imaging findings that could potentially discriminate these two conditions. The authors suggest that combining imaging and clinical features may be more helpful in achieving an accurate diagnosis rather than either alone. This report adds to a plethora of similar reports that suggest that, beyond a point, these findings may not help in achieving a conclusive diagnosis [10,11,12,13,14]. It remains to be seen whether the addition of upcoming armamentaria, like perfusion CT, or an even better application of simple techniques like intestinal bowel ultrasound would help in improving the differentiating ability [15,16].

Regarding inflammatory bowel disease (IBD), another study from Taiwan reported about 120 patients with IBD and Metabolic Dysfunction-Associated Fatty Liver Disease (MAFLD) in around 29% of patients. While, traditionally, primary sclerosing cholangitis (PSC) was considered the most important and consequential hepatobiliary manifestation of IBD, contemporary evidence suggests that MAFLD may now be a more common and important concern. The number of IBD cases is rising globally, including in the developing world [17]. This means that clinicians are more likely to deal with the extraintestinal manifestations and epiphenomena of IBD. A recent systematic review has suggested that the prevalence of fatty liver disease in IBD is 26.1% (95% CI: 22.1–30.2), while PSC is prevalent in 1.67% (95% CI: 1.47–1.88%) of cases [18]. The current study suggests a high prevalence in remission but lacks controls to conclusively confirm whether the prevalence is higher than in the general population. Nevertheless, clinicians treating IBD should be aware of underlying MAFLD in this population, and future work should also focus on fibrosis and noninvasive assessment in such a population [19,20].

A report by Ogasawara K and colleagues discusses the prediction of surgery in a small subset of newly diagnosed Crohn’s disease patients who were planned to undergo capsule endoscopy. Recent times have seen a growing interest in the role of surgery, and emerging evidence suggests that surgery may be a reasonable choice (as compared to biologicals) in limited disease [21,22]. However, often, the decision for surgery is influenced by the complications of underlying disease (strictures and fistulae) or medically refractory disease. In their small cohort, the authors suggest that small bowel patency, the extent of lesions, and age were predictors of need for surgery.

Another review from Harindranath S et al. focuses on the perplexing issues of differentiating a pancreatic adenocarcinoma from an inflammatory mass in the setting of chronic pancreatitis. This, indeed, is a difficult clinical situation where the yield of traditional fine-needle aspiration cytology is low due to underlying chronic pancreatitis-related fibrosis. Alarmingly, as authors suggest, a quarter of patients resected for seemingly non-neoplastic lesions can harbor malignancy, suggesting the need for a careful evaluation. The authors provide an algorithmic approach to deal with this clinical situation. 

Another review by Jearth V et al. deals with an ever-increasing problem of drug-resistant Helicobacter pylori. Helicobacter pylori is recognized not just as a cause of peptic ulcers but also as an agent implicated in gastric carcinogenesis. For this condition, the burden of disease is very high, especially in developing countries in Asia and Africa. Drug resistance is an ever-increasing threat, resulting in reduced cure rates with standard therapies and the need for costlier options [23,24]. This review provides a summary of the prevalence and mechanisms of drug resistance in Helicobacter pylori and also suggests molecular approaches to achieve early diagnosis.

Other papers in this issue deal with gastrointestinal oncology. In a study on 87 patients with pancreatic ductal adenocarcinoma, Rusu et al. studied the immunoexpression of Galectin-8 and noted its positivity in most (77%) cases. The authors suggest that nuclear labeling is associated with longer survival. The molecular classification of malignancies is an area of immense current interest and may offer targeted therapies in the future. The clarification of the clinical relevance of this biomarker, along with others, requires more work. In another report on 160 patients undergoing surgery for pancreatic cancer, Wang et al. suggest that higher cholesterol levels predict better survival. The authors collected data on serum cholesterol levels in the perioperative group at different periods. They found that total cholesterol levels at four weeks were independent predictors (long with tumor differentiation, pTNM stage, and lymph nodal metastasis) of long-term survival. This study provides insight into the use of a simple biomarker as a prognostic marker in postoperative pancreatic carcinoma. Further studies need to validate these findings in additional cohorts before clinical use can be considered.

In a report on 140 cases of primary gastrointestinal lymphoma collected over around three years, Tran QT et al. revealed that the most frequent site is the stomach, followed by the colon and small intestine. This study suggests a link between Helicobacter pylori and gastrointestinal lymphomas at all sites, including gastric MALTOMA. This study also describes the endoscopic features of various gastrointestinal lymphomas and provides a glimpse into the patterns of lymphomas seen in Vietnam.

Additionally, three brief papers report interesting cases: peculiar black esophagus (acute esophageal necrosis) in the setting of acute pancreatitis; a case of a malignant gastric neuroendocrine tumor in an elderly male; and appendiceal signet cell carcinoma presenting in the form of acute appendicitis in an elderly female.

Overall, the articles published in the current Special Issue suggest the need for better diagnostics and improved prognostic markers in the management of gastrointestinal diseases, including cancers. A stepwise algorithm-based approach may be of use in differentiating abdominal tuberculosis. The dilemma of making a positive diagnosis of abdominal tuberculosis is all too well known by clinicians in the developing world [25]. In our own experience, almost half of patients with Crohn’s disease have received empirical antitubercular therapy due to diagnostic confusion. Newer modalities are required to make a correct diagnosis, and CT perfusion appears promising for intestinal tuberculosis. Further work should focus on patients with an initial presentation in order to study its performance. Ascitic fluid analysis remains the modality of choice to discriminate tuberculous peritonitis and peritoneal carcinomatosis as the addition of clinical and radiological findings may not improve the discriminative value to a great extent [26]. Similarly, the evaluation of a pancreatic head mass in the setting of chronic pancreatitis should account for the fibrotic nature of the pancreatic malignancy and the false negativity of cytological evaluation. Furthermore, the search for prognostic markers in malignancy should also focus on relatively simple and cost-effective markers, as demonstrated in one of the studies. In conclusion, the articles featured in the present Special Issue are likely to aid towards better diagnosis and prognostication in gastrointestinal diseases.
